# General anesthesia as a possible GABAergic modulator affects visual processing in children

**DOI:** 10.3389/fncel.2013.00042

**Published:** 2013-04-23

**Authors:** C. Van den Boomen, J. C. de Graaff, T. P. V. M. de Jong, C. J. Kalkman, C. Kemner

**Affiliations:** ^1^Department of Experimental Psychology, Helmholtz Institute, Utrecht UniversityUtrecht, Netherlands; ^2^Department of Developmental Psychology, Utrecht UniversityUtrecht, Netherlands; ^3^Division of Anesthesiology, Intensive Care and Emergency Medicine, Department of Pediatric Anesthesia, University Medical Center/Wilhelmina Children's HospitalUtrecht, Netherlands; ^4^Department of Pediatric Urology, Pediatric Renal Center, University Medical Center/Wilhelmina Children's HospitalUtrecht, Netherlands; ^5^Academic Medical Center/Emma Children's HospitalAmsterdam, Netherlands; ^6^Department of Anesthesiology, Division of Anesthesiology, Intensive Care and Emergency Medicine, University Medical CentreUtrecht, Netherlands; ^7^Department of Child and Adolescent Psychiatry, Rudolf Magnus Institute of Neuroscience, University Medical CenterUtrecht, Netherlands

**Keywords:** GABA, development, general anesthesia, EEG, psychophysics, vision, segmentation

## Abstract

Gamma-Aminobutyric Acid (GABA) inhibitory interneurons play an important role in visual processing, as is revealed by studies administering drugs in human and monkey adults. Investigating this process in children requires different methodologies, due to ethical considerations. The current study aimed to investigate whether a new method, being general anesthesia using Sevoflurane, can be used to trace the effects of GABAergic modulation on visual brain functioning in children. To this aim, visual processing was investigated in children aged 4–12 years who were scheduled for minor urologic procedures under general anesthesia in day-care treatment. In a visual segmentation task, the difference in Event-Related Potential (ERP) response to homogeneous and textured stimuli was investigated. In addition, psychophysical performance on visual acuity and contrast sensitivity were measured. Results were compared between before and shortly after anesthesia. In two additional studies, effects at 1 day after anesthesia and possible effects of task-repetition were investigated. ERP results showed longer latency and lower amplitude of the Texture Negativity (TN) component shortly after compared to before anesthesia. No effects of anesthesia on psychophysical measurements were found. No effects at 1 day after anesthesia or of repetition were revealed either. These results show that GABAergic modulation through general anesthesia affects ERP reflections of visual segmentation in a similar way in children as benzodiazepine does in adults, but that effects are not permanent. This demonstrates that ERP measurement after anesthesia is a successful method to study effects of GABAergic modulation in children.

## Introduction

Gamma-Aminobutyric Acid- (GABA-)ergic inhibitory interneurons are crucial for brain functioning, as has been shown in psychopharmacological studies on visual processing in animals and human adults (e.g., Blin et al., 1993; Wang et al., 2002; van Loon et al., 2012). Both the GABAergic system in general as well as many aspects of visual processing are still maturing during childhood (Pinto et al., 2010; Kilb, 2012; van den Boomen et al., 2012). The role of GABA on visual processing during development remains, however, to be investigated. Studying this in human children is imperative, considering the proposed role of a GABAergic deficiency in developmental disorders such as Autism Spectrum Disorder and Schizophrenia, and the systematic occurrence of abnormalities in visual processing in these disorders (e.g., Hussman, 2001; Di Cristo, 2007; Yoon et al., 2010; Coghlan et al., 2012).

However, methods typically applied in adults to manipulate GABAergic processing, being administration of drugs such as benzodiazepines, can for ethical reasons not be used in children. In the current study, we used a new methodology and investigated the effects of GABAergic modulation in children who had to experience general anesthesia for surgery. Anesthesia was applied through inhalation of Sevoflurane. In the current study, interest was in this drug's GABA_A_-agonistic effect, which results in an increased and prolonged inhibition of neuronal transmission (Michel and Constantin, 2009). The aim of the present study was to investigate whether this method can be used to trace the effects of GABAergic modulation on visual brain functioning in children. Visual processing was measured before anesthesia administration and after anesthesia was terminated using Event-Related Potentials (ERP), a mobile, non-invasive and sensitive measure of brain activity that is suitable for children, and psychophysical measurements for behavioral differences.

**Figure 1:**
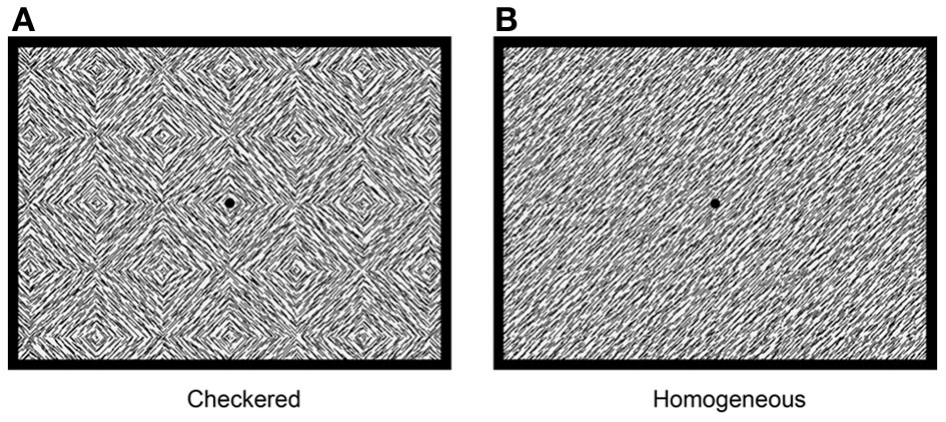
**Stimuli used for investigation of visual segmentation: checkered stimulus (A) and homogeneous stimulus (B)**.

For ERP measurements, the process of particular interest was visual segmentation, a process that allows for object perception. The process of visual segmentation includes integration of multiple elements of an object, detection of boundaries, and segregation of an object from its background or other objects, and is thought to be dependent on feedback and horizontal connections operating via GABAergic interneurons in the visual cortex (Roelfsema et al., 2002; Scholte et al., 2008). Visual segmentation is typically investigated by comparing brain activity evoked by two types of stimuli: one containing borders or figures, such as checkered (textured) stimuli (Figure 1A), vs. homogeneous stimuli (Figure 1B). Both stimuli are made using the same elements, but they differ in the level of visual segmentation required to process them. Brain activity is modulated by the checkered but less so by homogeneous stimuli at latencies beyond 100 ms. after stimulus onset (e.g., Lamme, 1995; Zipser et al., 1996; Lamme et al., 1998a; Super et al., 2001). This modulation is evident as a negative peak in the difference wave in ERPs evoked by textured vs. homogeneous stimuli (e.g., Bach and Meigen, 1992, 1998; Lamme et al., 1992; Caputo and Casco, 1999; Scholte et al., 2008), which is referred to as Texture Negativity (TN). Previous psychopharmacological research in human adults revealed that visual segmentation was affected by GABAergic modulation (e.g., Giersch et al., 1997; Giersch and Lorenceau, 1999; Beckers et al., 2001; Elliot et al., 2006; van Loon et al., 2012). Effects were absent after NMDA or muscarinic modulation, which suggests specific involvement of GABA in this process (van Loon et al., 2012). Therefore, checkered and homogeneous stimuli can be used to investigate whether GABAergic modulation specifically affects brain processes involved in visual segmentation in children.

**Figure 2:**
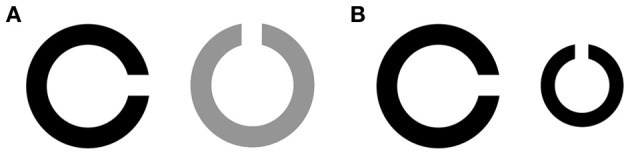
**Examples of stimuli for investigation of contrast sensitivity (A) and visual acuity (B)**.

Using psychophysical measurements, we investigated effects on two other aspects of visual function, namely contrast sensitivity (i.e., ability to discriminate different luminance levels of two connecting parts of a visual stimulus, Figure 2A) and visual acuity (i.e., sharpness of vision, Figure 2B). Of these processes, contrast sensitivity was diminished after GABAergic modulation in adult psychopharmacological studies, while visual acuity remained intact (Blin et al., 1993; Speeg-Schatz et al., 2001; Giersch et al., 2006a). The specific mechanisms underlying these findings remain unknown. Proposals on this matter include a diminished sensitivity to light due to effects at a retinal level, or a modulation of specific neural pathways throughout the visual cortex (see Giersch et al., 2006a, for an in-depth discussion). Nevertheless, multiple studies did reveal specific effects of GABAergic modulation on these rather basic visual processes, which could influence possible effects on visual segmentation. Therefore, these tasks can be used to investigate the specific psychophysical effects, being diminished contrast sensitivity and non-affected visual acuity, of GABAergic modulation through anesthesia in children and control for an influence of these effects on visual segmentation.

Effects of GABAergic modulation were investigated at two time-points in separate studies. In study 1, effects on visual processing were investigated during the recovery phase after anesthesia. Sevoflurane concentration at exhalation was measured after anesthesia to investigate whether possible effects on visual processing were acute or indirect (i.e., measured when anesthetics were either still present or eliminated from the system). Note that measurement after elimination would allow for an extension of the acute investigations that are typical in adult psychopharmacological research. In study 2, we investigated whether effects of GABAergic modulation were still present after 1 day. Previous studies have suggested that such modulation could affect visual processing for several hours in adults (Blin et al., 1993) and that anesthesia could have permanent effects on brain functioning in children (Wilder et al., 2009). Study 2 could shed more light on the permanency of possible effects of anesthesia on vision in children. To control for repetition effects, in a control study (study 3) the effects of task-repetition on ERP peaks reflecting visual segmentation were investigated.

## Materials and methods

### General methods

In total 36 subjects were recruited to participate in study 1, 16 subjects in study 2, and 9 in study 3. Subjects in study 1 and 2 were patients at the department of child-urology of the tertiary children's university hospital (Wilhelmina Child Hospital, University Medical Center, Utrecht, Netherlands), who were scheduled for urologic procedures under general anesthesia with monotherapy Sevoflurane (>30 min) with locoregional analgesia (caudal block with marcaine) in day-care treatment. Subjects did not suffer from any neurological disease or psychiatric disorder and had normal or corrected to normal vision. The study was approved by the medical ethics committee of the University Medical Centre Utrecht. Parents of the subjects gave written informed consent prior to participation. Subjects were rewarded with a toy after participation. All subjects participated in two sessions, a pre-session before and a post-session after anesthesia, of which the time-points differed between study 1 and 2. Subjects in study 3 were healthy children, not scheduled for surgery, and recruited through primary schools in the Netherlands.

## Study 1 – short-term effects

### Methods

#### Procedure

Subjects participated in two sessions. The first session (pre-test) took place within 2 h before surgery, and the second (post-test) took place as soon as possible after anesthesia, during recovery. Both sessions took place in the same separate, quiet and luminance-controlled room at the post-operative recovery unit of the tertiary children's university hospital. The same task-procedure was applied in both sessions: first the visual acuity and contrast sensitivity tasks were performed in counterbalanced order, followed by the visual segmentation task.

Children were anesthetized through inhalation induction with Sevoflurane (8%) by face mask. After a peripheral venous line for intravenous fluid (ringers lactate 10 ml/kg/h) was applied, spontaneous breathing through laryngeal mask airway was maintained with an inspiratory concentration of Sevoflurane of 2.5%. An epidural or caudal infiltration (1–1.25 ml/kg solution of marcaine 1.75 mg with epinephrine 0.0025 mg/ml) was applied for perioperative and post-operative analgesia. Except for propofol (*n* = 3; 2.5–6 mg/kg), lidocaine (*n* = 2; 0.5–1 mg/kg) and fentanyl (*n* = 1; 0.1 mcg/kg) at induction, pain prophylaxis (intravenous administration of acetaminophen 15 mg/kg and/or voltaren 1 mg/kg) or antiemetics (ondasteron *n* = 4; 0.1 mg/kg or dexamethasone *n* = 1; 0.1 mg/kg) no other medication were administered before the tasks were finished.

Post-test took place when children were awake and free of pain and nausea. During the full recovery phase oxygen saturation and heart rate were routinely measured. Additionally, expiratory Sevoflurane concentration was measured through a nasal sampler during the tasks in the post-test phase in all children, and during the total recovery phase in 6 of the included participants to validate the measurement.

#### Tasks

Visual acuity was measured using the Freiburg Visual Acuity and Contrast Test (FrACT, version 3.3), which is a validated and freely available digital version of the standard Landholt-C chart (Bach, 1996). In this task, a black circle of various sizes is presented on a white background (Figure 2B). The circle contains a gap at one out of four possible sites. Task of the participant is to indicate the position of the gap by pointing or naming. A total of 30 trials are presented. Threshold results are calculated into Snellen and Vernier Acuity, of which Snellen Acuity will be presented in this study.

Contrast sensitivity was also measured using the FraCT (Bach, 1996). In this task, circles were of various luminance contrasts (Figure 2A). Threshold results are calculated into Michelson and Weber Contrast, of which Michelson Contrast will be presented in this study.

Brain activity was measured during the visual segmentation task. Two types of stimuli were presented: homogeneous (eight different stimuli) and checkered stimuli (also eight different stimuli, see Figure 1 for an example). Stimuli consisted of 45 or 135° oriented black line segments, randomly positioned on a white background (total 450 line segments; 100% luminance contrast). The lines were organized either into homogeneous or into checkered stimuli such that local modulations are, on average, equally distributed between the two types of stimuli. However, due to the homogeneous or checkered fashion in which stimuli were presented, the amount of segmentation was modulated independently of the changes in orientation that occur on each transition from one stimulus to the next (see Lamme et al., 1992 for a full explanation of this balancing of local modulation). Stimuli subtended 17.5 × 13° of visual angle at a viewing distance of 57 cm. Homogeneous and checkered stimuli were alternated every 800 ms. The basic sequence consisted of the 16 stimuli presented in a fixed order. A total of 400 stimuli were presented (200 per condition). Participants performed an oddball task on Pokemon cartoons presented at random time-points during the task.

#### EEG recording and analyses

During the visual segmentation task, brain activity was recorded using Biosemi Active Two EEG system (Biosemi, Amsterdam, Netherlands). For both studies, vertical EOG was recorded from electrodes placed above and below the left eye, and horizontal EOG from electrodes placed at the outer canthi of the eyes. Brain activity was recorded from 1 electrode (Oz), based on previous research (Lamme et al., 1992). Two additional electrodes were placed at the left and right mastoid, and provided an active ground that was used as a reference point. These were the Common Mode Sense (CMS; placed on left mastoid) and Driven Right Leg (DRL; placed on right mastoid). During recording, EEG was sampled at a rate of 2048 Hz.

**Figure 3:**
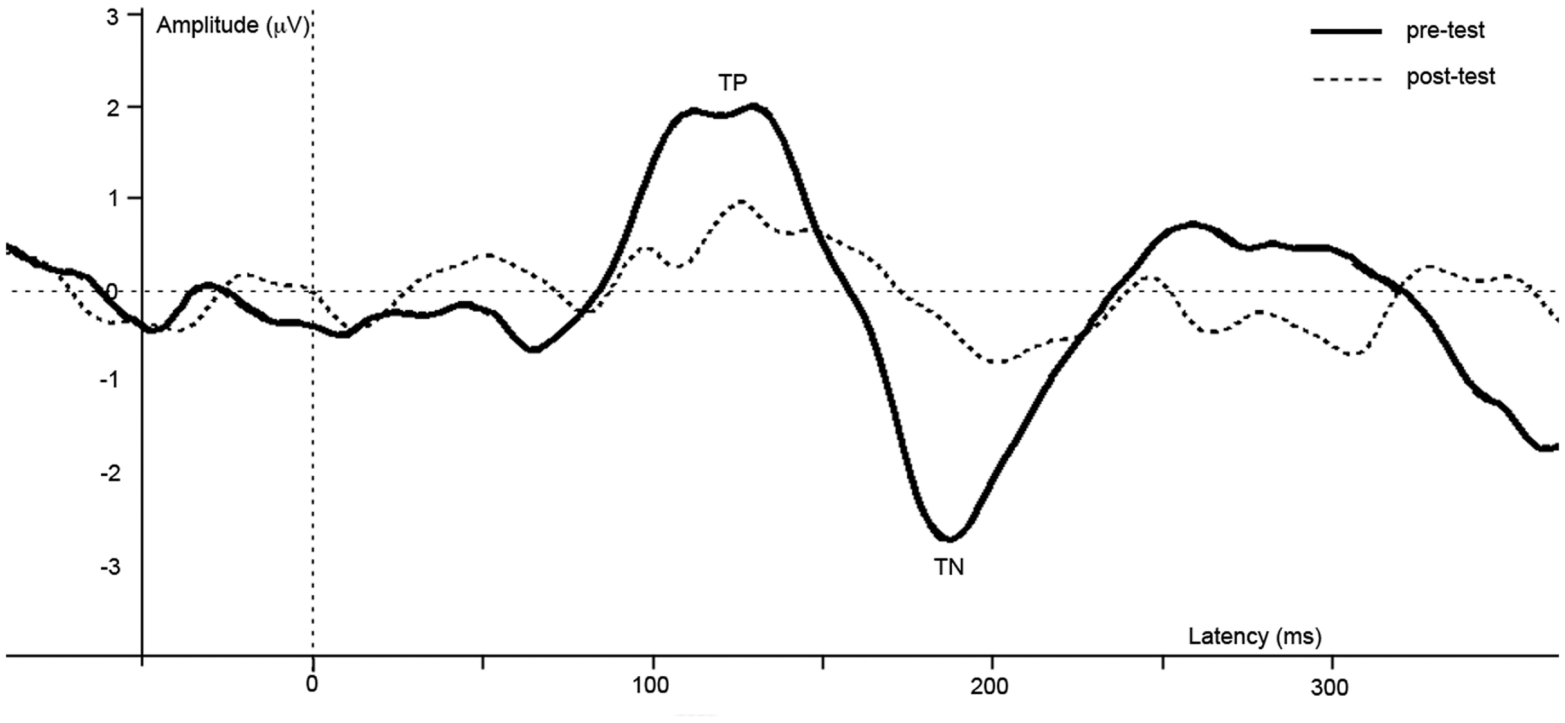
**Difference waves of grand averages at Oz electrode evoked by checkered vs. homogeneous stimuli in the pre-test (solid line) and post-test (dashed line) of the short-term study**.

All pre-processing and visualization steps were done using Brain Vision Analyzer (Brain Products GmbH, Munich, Germany). After recording, data were re-sampled to 512 Hz, and filtered with a high-pass filter of 1 Hz, a low-pass filter of 30 Hz and a notch filter of 50 Hz. In order to compute ERPs, epochs of 50 ms pre-stimulus (baseline) until 400 ms post-stimulus were extracted from the continuous data. Ocular artifacts were removed from the EEG with a regression analyses based on eye-movements detected by vertical EOG (blinks) and horizontal EOG electrodes (horizontal eye-movements) (Gratton et al., 1983). Epochs with additional amplitude artifacts were removed. These artifacts were defined by a voltage change of 50 μV per sampling point, a difference of 3 μV per 200 ms, or amplitudes below −100 or above 100 μV. These values are based on previous research using the same task in children (Kemner et al., 2007). ERPs were averaged separately for the checkered and homogeneous stimuli. Next, subtracting the ERP evoked by the homogeneous from that evoked by the checkered stimuli created a difference wave. Peaks in this difference wave are thought to reflect the modulation of brain activity related to visual segmentation. In the difference wave a negative peak (referred to as TN) is typically detected beyond a latency of 100 ms. after stimulus onset in adults (e.g., Lamme, 1995; Zipser et al., 1996; Lamme et al., 1998a; Super et al., 2001). In children, an additional positive peak was apparent as well, which preceded the TN (see Figure 3). This peak will be referred to as texture positivity (TP). For the pre-test, the TP was scored as the most positive point (global maxima) directly preceding the TN. Scoring was performed semi-manually to prevent miss-detection of possible positive peaks following the TN. The TN was automatically scored as the most negative point (global maxima) between 130 and 190 ms post-stimulus. For the post-test, the TP was scored as the global maxima in a time-interval of 29 ms (*SD* + 20 ms) before or after the TP in the pre-test of the individual. The TN was scored as the global maxima in a time interval of 32 ms (*SD* + 20) before or after the TN in the pre-test of the individual. Four paired *t*-test were done using PASW 18 (SPSS Inc., Chicago, IL, USA), with TP and TN latency or amplitude as dependent variables, and anesthesia (pre vs. post) as independent variables. For all reported analyses, *t*-tests were two-sided and the alpha value was set at 0.05.

#### Psychophysical analyses

The effects of anesthesia on visual acuity and contrast sensitivity were analyzed using two paired *t*-test, comparing pre- and post-anesthesia thresholds.

### Results

#### Included participants

Of the 36 recruited subjects, 19 subjects (12 males) were included in the analyses for visual segmentation (EEG brain activity measurement; average age 7.5 years; *SD* 1.8) and 21 subjects (14 males) were included in analyses for visual acuity and contrast sensitivity (psychophysical measurement; average age 7.4 years; *SD* 2.0).

Participants were excluded from both psychophysical and EEG analyses because of change in anesthesia procedure (*N* = 6) and no post-test (*N* = 1). In addition, participants were excluded from EEG analyses because of change in design (*N* = 5; the change included presentation of more stimuli due to low signal-to-noise ratio in the first five participants), no pre- and post-test due to shortage of time (*N* = 1), no post-test (*N* = 3), and too low attention (*N* = 1). For the psychophysical tasks, additional participants were excluded from analyses because of technical error (*N* = 1), no pre- and post-test due to shortage of time (*N* = 4), and no post-test (*N* = 3). Reasons for absence of post-test were nausea, tiredness, and no interest in participation.

Average duration of anesthesia of the included participants was 51 min (*SD*: 15), with average concentration of inhaled Sevoflurane (IT) of 3.165% (*SD*: 0.392). Expired concentration of Sevoflurane (ET) were measured at start of recovery monitoring in six children, on average 4.7 min after termination of anesthesia. In three children, Sevoflurane concentration was already decreased to zero at start of monitoring. In the other three children, the expiratory concentration of Sevoflurane was on average 2.67% at start of monitoring and decreased to zero within 8–28 min after termination of anesthesia. Participants performed the contrast sensitivity, visual acuity, and visual segmentation tasks, respectively, at on average 40, 41, and 55 min after anesthesia was ended. At this time, Sevoflurane exhalation values were returned to zero in all participants, conform reported Sevoflurane elimination kinetics in children (Landais et al., 1995).

#### EEG

ERPs of grand average difference waves are presented in Figure 3, an overview of test-results is provided in Table 1. Paired *t-tests* revealed no significant effect of anesthesia on TP latency or amplitude. Paired *t-tests* revealed a significantly longer latency and lower amplitude of TN after compared to before anesthesia.

**Table 1:** **Overview of EEG and psychophysical results of study 1, 2, and 3**.

**Measurement**	**Pre-test mean ± *SE***	**Post-test mean ± *SE***	***t*-value (df)**	***p*-value**
**STUDY 1 – SHORT-TERM**				
TP amplitude	3.0 ± 0.6	2.7 ± 0.7	0.40 (18)	0.702
TP latency	131 ± 8.5	139 ± 11.2	–1.74 (18)	0.099
TN amplitude	−4.9 ± 1.0	−2.9 ± 0.7	–2.46 (18)	0.024^*^
TN latency	196 ± 12.1	205 ± 11.7	–2.42 (18)	0.027^*^
Acuity	15.6 ± 0.84	16.1 ± 0.93	–0.92 (20)	0.37
Contrast	1.1 ± 0.06	1.1 ± 0.10	–0.75 (20)	0.46
**STUDY 2 – LONG-TERM**				
TP amplitude	2.4 ± 0.9	2.2 ± 0.5	0.31 (7)	0.767
TP latency	130 ± 6.0	134 ± 7.6	–0.48 (7)	0.646
TN amplitude	−3.6 ± 0.7	−3.4 ± 1.0	–0.19 (7)	0.853
TN latency	187 ± 5.5	182 ± 7.7	0.56 (7)	0.596
Acuity	18.6 ± 1.8	19.6 ± 2.3	–0.53 (12)	0.603
Contrast	1.3 ± 0.44	1.5 ± 0.49	–0.76 (12)	0.463
**STUDY 3 – CONTROL**				
TP amplitude	4.7 ± 0.6	3.8 ± 0.7	0.79 (8)	0.455
TP latency	131 ± 9.2	124 ± 5.6	0.80 (8)	0.446
TN amplitude	−0.8 ± 0.8	−2.1 ± 0.6	1.46 (8)	0.182
TN latency	201 ± 11.3	198 ± 12.8	0.59 (8)	0.573

Asterisks indicate significant effects.

#### Psychophysical performance

No effects of anesthesia on visual acuity and contrast sensitivity were found.

### Conclusions study 1 – short-term effects

In the present study, we investigated the effects of GABAergic modulation through general anesthesia on visual processing in children directly after surgery. Results revealed that brain activity related to visual segmentation was diminished and slower compared to before modulation. No effects on psychophysical measurements of visual acuity and contrast sensitivity were found. To investigate whether these effects remained until one day after surgery, long-term effects were tested in study 2. To control for possible effects of task-repetition we conducted a control study (study 3), in which children performed the task twice in a row without modulation.

## Study 2 – long-term effects

### Methods

Methods in study 2 are equal to those in study 1 except of the below described discrepancies.

#### Procedure

For investigation of long-term effects, the pre-test took place in the week prior to surgery, and the post-test at 1 day after surgery. Both sessions took place in a quiet room at children's home. In both sessions, two additional visual tasks were performed to measure spatial frequency processing and contour integration, of which methods and detailed description of results (no effects of anesthesia; all *p*-values above 0.05) are not reported here.

#### EEG recording and analyses

Brain activity was recorded from 32 electrodes, positioned at standard EEG recording locations according to the 10/20 international system. The CMS and DRL electrodes were located in the cap, and two additional electrodes were placed at the left and right mastoid for offline re-referencing purposes.

In both the pre- and post-test the TP was semi-manually scored as the most positive point (global maxima) directly preceding TN, which was automatically scored as the most negative point (global maxima) between 130 and 190 ms post-stimulus.

### Results

#### Included participants

Of the 16 recruited subjects, 8 (7 males) were included in the analyses for visual segmentation (EEG brain activity measurement; average age 8.4 years; *SD* 2.6) and 13 subjects were included in analyses for visual acuity and contrast sensitivity (psychophysical measurement; average age 8.1 years; *SD* 2.0).

Participants were excluded from both psychophysical and EEG analyses because of psychiatric disorder (*N* = 1) and no post-test (*N* = 1). In addition, participants were excluded from EEG analyses because of change in design (*N* = 4), no post-test for EEG (*N* = 1), and technical error (*N* = 1). For the psychophysical tasks, additional participants were excluded from analyses because of no pre- and post-test due to shortage of time (*N* = 1).

Average duration of anesthesia of the included participants was 50 min (*SD*: 23), with average Sevoflurane inspiration concentration of 3.173% (*SD*: 0.558). Duration and inspiration concentration are not significantly different from those in study 1 (independent *t*-tests, *p* > 0.05).

#### EEG

**Figure 4:**
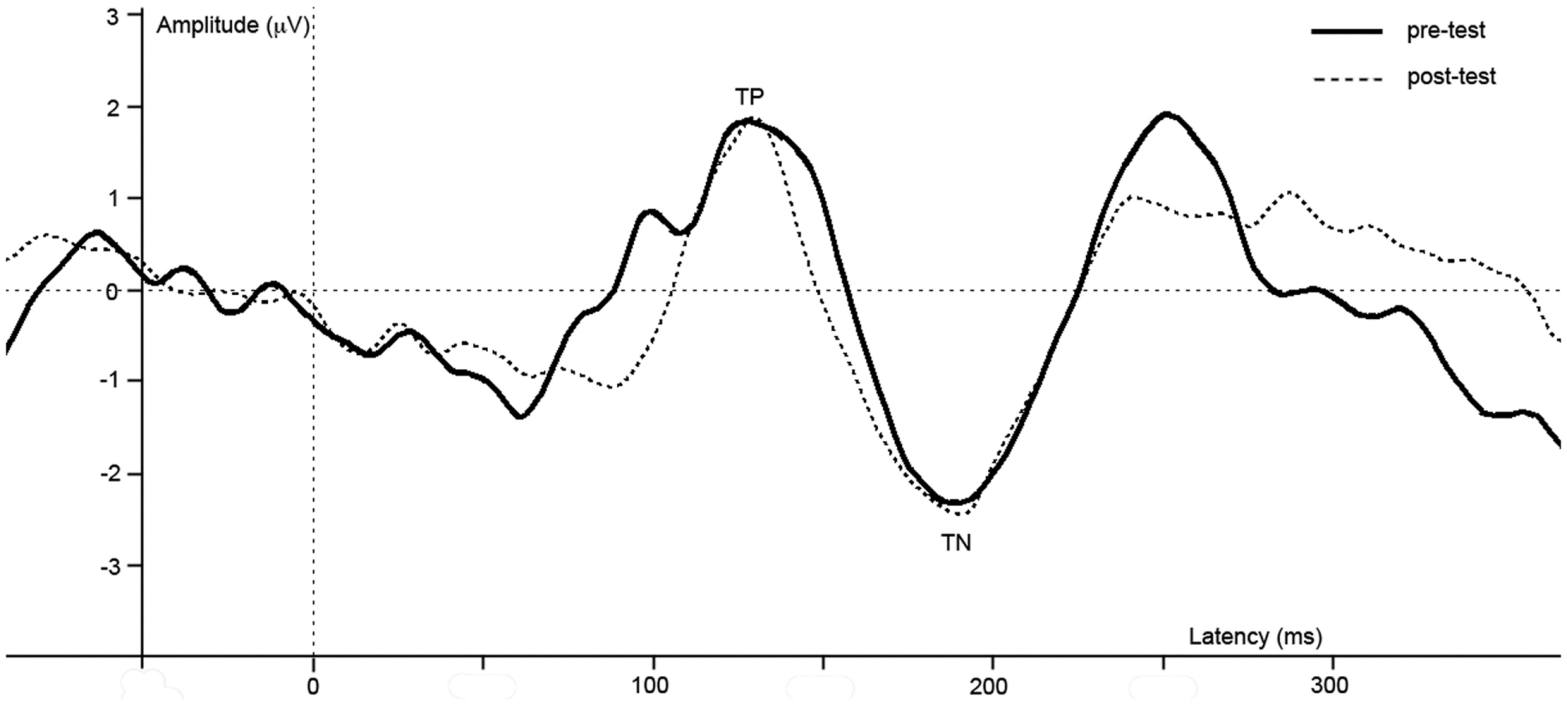
**Difference waves of grand averages at Oz electrode evoked by checkered vs. homogeneous stimuli in the pre-test (solid line) and post-test (dashed line) of the long-term study**.

ERPs of grand average difference waves are presented in Figure 4, an overview of test-results is provided in Table 1. Paired *t*-tests revealed no significant effect of anesthesia on TP or TN latency or amplitude.

#### Psychophysical performance

No effects of anesthesia were revealed on visual acuity or contrast sensitivity.

### Conclusions study 2 – long-term effects

In the current study, we investigated whether effects of GABAergic modulation through general anesthesia on visual processing in children remained to be present at 1 day after surgery. Results showed no difference in brain activity or psychophysical measurements of visual processing after compared to before surgery, which indicates that the reported effects directly after anesthesia have deteriorated after 1 day.

## Study 3 – control study for repetition effect

### Methods

A control study was conducted to investigate effects of task-repetition on ERP amplitude and latency in healthy children. Methods in study 3 are equal to those described for study 2 except of the below described discrepancies.

#### Participants

Nine subjects (all males) participated in this study (all males; average age 8.3 years; *SD*: 0.23). All subjects were included in the analyses for EEG analyses and no psychophysical investigation of visual acuity and contrast sensitivity was performed.

#### Procedure

Children participated in one session in which the visual segmentation task was performed twice in a row without interruption of anesthesia. Testing took place in a quiet room at primary schools, at children's home, or at the University Medical Centre Utrecht.

### Results

#### EEG

**Figure 5:**
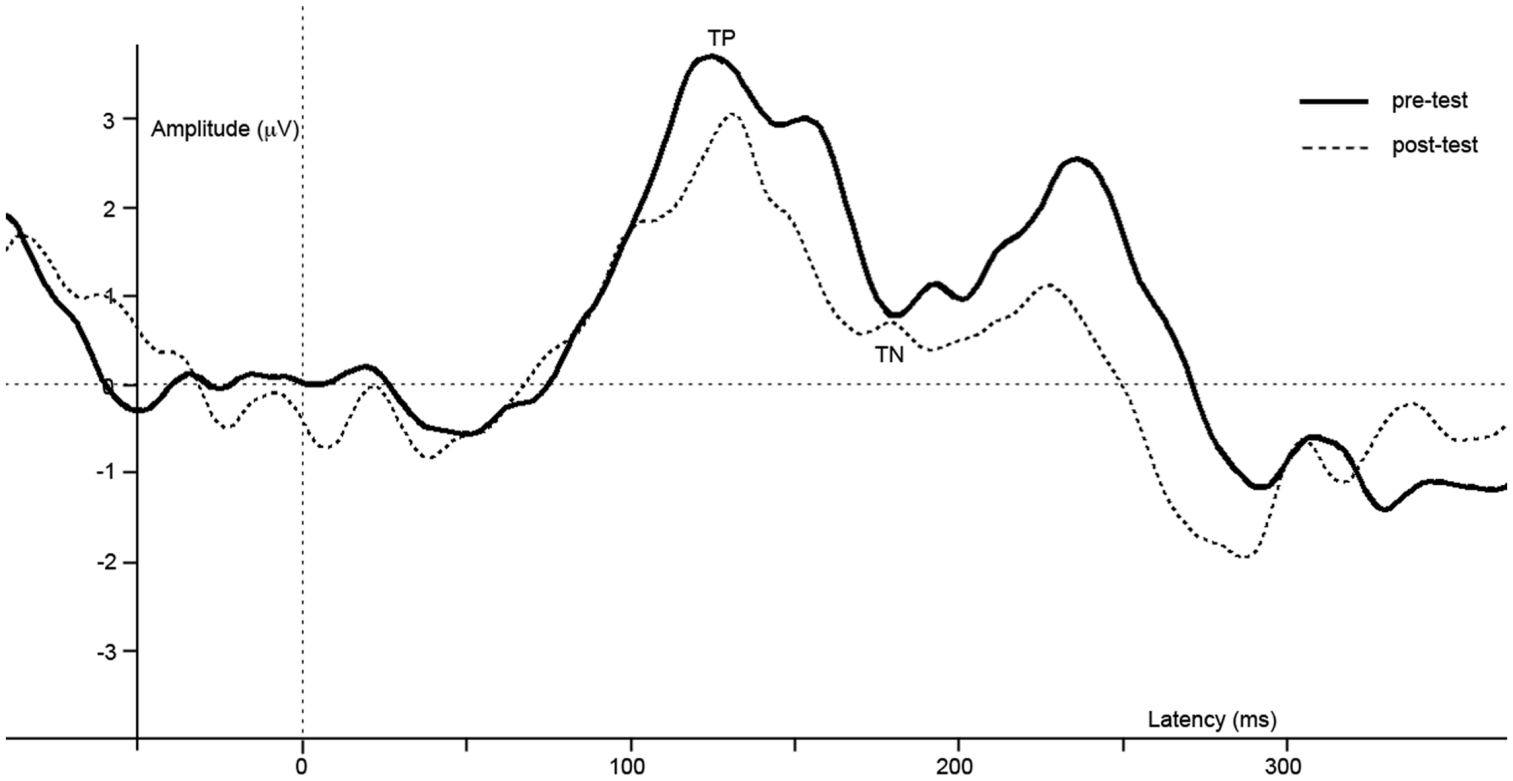
**Difference waves of grand averages at Oz electrode evoked by checkered vs. homogeneous stimuli in the pre-test (solid line) and post-test (dashed line) of the control study**.

ERPs of grand average difference waves are presented in Figure 5, an overview of test-results is provided in Table 1. Paired *t*-tests revealed no significant effect of repetition on TP or TN latency or amplitude.

### Conclusions study 3 – control study for repetition effect

In this control study, we investigated whether effects reported in study 1 could be due to repetition of the task. Results reveal no difference in brain activity between the first and the second run of the task, which implies the reported effects in study 1 are not due to task-repetition.

## General discussion

In the present study, we used general anesthesia as a new method to study the effects of GABAergic modulation on visual processing in children. For this purpose, we investigated several visual functions including visual segmentation, acuity and contrast sensitivity in children before and after anesthesia through inhalation of Sevoflurane, applied for urological surgery. ERP peaks reflecting visual segmentation showed a lower amplitude and longer latency directly after anesthesia, but no measurable psychophysical effects were present for contrast sensitivity and visual acuity. In addition, no effects of GABAergic modulation on visual processing were present 1 day after anesthesia.

The lower amplitude of ERP peaks that reflect visual segmentation (referred to as TN), present directly after anesthesia in children, is comparable to effects on ERP reported after benzodiazepine administration in adults (van Loon et al., 2012). The lower amplitude represents a smaller difference in brain activity evoked by the presented checkered vs. homogeneous stimuli. The current data do not allow for solid conclusions on the specific mechanisms underlying this smaller difference. However, based on the literature some suggestions can be proposed. The TN is often related to an increased activity in horizontal and feedback neural connections evoked by the checkered vs. homogeneous stimuli, proposed to lead to detection of boundaries and perceptual grouping, respectively (Roelfsema et al., 2002; Scholte et al., 2008). Furthermore, these connections function, among others, via GABAergic inhibitory interneurons (Iversen et al., 1971; Roelfsema et al., 2002). Although the specific neuro-chemical mechanisms underlying the decreased TN cannot be inferred from ERP measurements, the effects supposedly result from a GABAergic modulation of horizontal and feedback connections in the visual cortex. In addition, the TN showed a longer latency after anesthesia. This effect suggests delayed visual processing, an effect of GABAergic modulation that is consistent with the results of a psychophysical masking task in adults (Giersch and Herzog, 2004). These results show that effects of GABAergic modulation can be indexed through the TN in children. The effective use of ERP measurements after anesthesia as a method to trace the effects of GABAergic modulation has imperative implications for future research on the role of GABAergic deficiencies in developmental disorders. Using this new method, the role of GABA in other sensory and cognitive processes in the developing brain can now be investigated.

Regarding psychophysical measurements of contrast sensitivity and visual acuity, results of the current study do not match the previously reported decrease in contrast sensitivity in adults (Blin et al., 1993; Speeg-Schatz et al., 2001; Giersch et al., 2006b). Multiple explanations for the absence of effect are possible. It could be that contrast sensitivity was decreased during and very shortly after anesthesia, when anesthetics were present in the body (see also the discussion below), but had recovered at the moment of measurement. An alternative explanation is that GABAergic modulation affects contrast sensitivity in a different way in children compared to adults, due to the still underdeveloped visual system at the age of measurement (van den Boomen et al., 2012). Furthermore, although Sevoflurane and benzodiazepines are both GABA_A_-antagonists, Sevoflurane has also a NMDA antagonistic effect. The difference in mechanisms of action on various receptor subtypes might contribute to the difference in observations between adults and children (Grasshoff et al., 2006; Olkkola and Ahonen, 2008; Michel and Constantin, 2009).

There is a difference between the studies in adults using benzodiazepines and our study, with regard to the time of measurement in relation to modulation. In adults acute effects on visual processing are studied, as experimental measurements are being done during the effective stage of benzodiazepines. In the current study, however, the exhalation data show that there were no more anesthetics (Sevoflurane) to a measurable extent present at expiration at the moment of testing. This might imply that Sevoflurane itself was not present in the brain. Importantly, this shows that GABAergic modulation during a relatively short time (average 51 min) in children has a sustained effect on brain activity that can be seen *after* modulation. These effects do, however, deteriorate, as shown by their absence at 1 day after modulation.

Importantly, other aspects that could explain the current ERP results should be considered. One is the possible effect of remaining sedative state or tiredness on visual processing. The absence of an effect of anesthesia on psychophysical tasks does, however, demonstrate that children were able to participate and attend to the stimuli. Furthermore, previous research shows no correlation between subjective sedation and effects of GABAergic modulation on ERPs or psychophysical measurements in adults (Giersch et al., 2006a; van Loon et al., 2012). This indicates that the ERP effects in the current study are not likely to be due to a change in sedative state. Another issue is the repetition of task after a relatively short delay (i.e., after a few hours and within a week, in study 1 and 2, respectively), which could have affected the ERP amplitude. To control for this, we have conducted an additional study (study 3) in which healthy children performed the visual segmentation task twice in a row. The absence of a difference in ERP peaks in this control study proves that the diminished amplitude directly after anesthesia is not due to repetition of task. Moreover, modulatory effects of anesthesia on low-level visual processing, as reported in previous studies (Blin et al., 1993; Speeg-Schatz et al., 2001; Giersch et al., 2006a), would affect segmentation as well. Important processes for segmentation based on changes in orientation of black lines are visual acuity, contrast sensitivity, and orientation processing. Visual acuity and contrast sensitivity were not affected in the current study. Furthermore, a previous study in monkeys revealed that anesthesia did not affect orientation processing, while reducing visual segmentation (Lamme et al., 1998b). These results indicate that the ERP effects are not due to changes in these low-level visual processes.

A last aspect to consider is whether the reported ERP effects are specifically due to GABAergic modulation as opposed to other neurotransmitter systems: although the GABA_A_-receptor is a main target of Sevoflurane, other neurotransmitter systems are modulated as well (Michel and Constantin, 2009). However, previous research in adults revealed that a lower amplitude of ERP peaks was present after specific GABAergic targeting, but absent after modulation of other systems (NMDA and muscarinic; van Loon et al., 2012). Thus, although (interaction-) effects of other systems can in psychopharmacological research never be fully excluded, comparison with adult research suggests a specificity of GABAergic modulation on the current ERP task.

In conclusion, to our current knowledge this is the first study showing the effects of anesthesia as a method for GABAergic modulation on visual processing in children, and that these can be assessed through ERPs. This new method paves the way for further research on the role of GABA on cognitive processing in the developing brain, increasing our understanding of the biological bases of neurodevelopmental disorders.

### Conflict of interest statement

The authors declare that the research was conducted in the absence of any commercial or financial relationships that could be construed as a potential conflict of interest.
